# Study on the Effect of Solution Conditions on Heat Induced-Aggregation of Human Alpha Interferon 

**Published:** 2014

**Authors:** Faranak Salmannejad, Nastaran Nafissi-Varcheh, Alireza Shafaati, Reza Aboofazeli

**Affiliations:** a *Department of Pharmaceutics, School of Pharmacy, Shahid Beheshti University of Medical Sciences, Tehran, Iran.*; b *Department of Pharmaceutical Biotechnology, School of Pharmacy, Shahid Beheshti University of Medical Sciences, Tehran, Iran.*; c *Department of Pharmaceutical Chemistry, School of Pharmacy, Shahid Beheshti University of Medical Sciences, Tehran, Iran. *

**Keywords:** Interferon alpha2b, Thermal aggregation, SE-HPLC, Optical density, SDS-PAGE

## Abstract

A major problem in the formulation of therapeutic proteins is the irreversible protein aggregation. Recombinant human interferon alpha2b (rhIFNα2b) has poor stability and undergoes physical degradation. The aim of this study was to investigate the effect of solution conditions on the heat-induced aggregation of rhIFNα2b. The protein was incubated for 1 h at 40–70 °C and for up to 240 h at 50 °C and its aggregation tendency was then studied using optical density (at 350 nm), SE-HPLC, dynamic light scattering and SDS-PAGE methods. The effect of various pH (5, 6 and 7) and buffer concentrations (10, 55 and 100 mM) on the aggregation of protein following incubation at 50 °C for 72 h was also evaluated. The results obtained for samples incubated at 50 °C for up to 240 h showed that OD350 and the amount of higher molecular weight aggregates (HMW) increased and the monomer content decreased significantly (p<0.05) as the incubation time increased. Following incubation at various temperatures, a significant increase in OD350, drop in monomer content and increase in the amount of HMW aggregates were observed (p<0.05). Data obtained from incubation of samples at 50 °C for 72 h confirmed that regardless of the buffer concentration, the percentage of monomer at pH 6 was significantly higher than that at pH 7 and pH 5 (p<0.05). At constant pH, although not significant, the same trend was observed when the buffer concentration increased to 100 mM. In conclusion, the change in solution conditions can influence the aggregation extent of rhIFNα2b.

## Introduction

During the past three decades, many therapeutic proteins have been developed for a wide range of human disorders (1). However, the main impediment for their commercialization has been to design and develop a formulation with an acceptable long shelf life. The problem arises from the inherent instability and molecular complexity of proteins (2, 3). Thus, a major challenge for a biopharmaceutical product formulator is to stabilize the molecule and postpone its degradation procedures. It is particularly difficult to achieve this goal, due to the protein susceptibility to chemical and physical degradation. In general, degradation causes a loss of protein product quality and, critically, may lead to a decrease of safety and efficacy and occurrence of adverse effects. Aggregation is one of the main physical instabilities of proteins which is recognized as a critical degradation pathway with clear influences on the efficacy and safety of a biological product and may be a potent inducer for immune responses with varying manifestations (4, 5). Aggregates are typically defined as assemblies of protein molecules, the level of which increase with time of storage and/or in response to stress.

Several types of stresses (*e.g*, thermal, acidic, mechanical, and interface adsorption) that proteins may face during the various production and manufacturing processes (*i.e*, expression, purification and formulation) have been identified and reported in the literature. In these processes, proteins are exposed to changes of the properties of the solution environment such as pH, temperature, salt concentration, buffer composition, surfactants, preservatives, shear rate and surfaces (6-9), which may influence the protein stability and facilitate their aggregations.

Interferons (IFNs) are cytokines with immunomodulatory, anti-proliferative and antiviral properties (10, 11). Specifically, 23 species of structurally similar proteins with molecular masses varying from 17 to 28 kDa have been identified in the human alpha interferon family (12). Among these subtypes, recombinant human IFN alpha2a (rhIFNα2a) and IFN alpha2b (rhIFNα2b) are the most successfully commercialized drugs (13) and used for the treatment of a variety of diseases such as hairy cell leukemia, multiple myeloma, basal cell carcinoma, and hepatitis B and C (14, 15). However, development of stable IFNα formulations still remains a great challenge (16). There are some reports on aggregation of IFNα products that could subsequently enhance their immunogenicity (17, 18). Sharma *et al. *have reported that IFNα2 is likely to form partially unfolded intermediates with conformations which are sensitive to solution pH and temperature. They showed that these unfolded states could play important roles in the aggregation of IFNα2 and put long-term stability of the protein at risk (6). The aim of this study was to investigate the influences of solution conditions on the aggregation behavior of rhIFNα2b. For this purpose, the aggregation tendency of rhIFNα2b was studied and evaluated at different temperatures, pH values and buffer concentrations, using various analytical methods.

## Experimental

*Materials*

rhIFNα2b was kindly gifted by Pasteur Institute of Iran (Tehran, Iran). Acrylamide/bis acrylamide solution, ammonium peroxodisulfate, bromophenol blue sodium salt, Coomassie brilliant blue, 1,4-dithiothreitol (DTT), glycerol, glycine, iso-butanol, 2-mercaptoethanol, sodium chloride, sodium dodecyl sulfate (SDS), di-sodium hydrogen phosphate anhydrous, sodium dihydrogen phosphate 1-hydrate, tetramethylethylenediamine (TEMED), and tris (hydroxyl methyl) amino methane (Tris) were purchased from Merck (Darmstadt, Germany). All solutions were prepared with sterile deionized water (Millipore Company, USA). Buffers were filtered through 0.2 μm membranes prior to use. 

*Stress studies*

The thermal stability of rhIFNα2b was investigated following heating the protein samples. Protein samples composed of 100 μg/mL in 100 mM sodium phosphate buffer solution (pH 7.0) were incubated (IFE 500, Memmert, Germany) for 1 h at various temperatures (40, 50, 55, 60, 65 and 70 °C) and then cooled to room temperature. The level of heat-induced aggregation and monomer content were monitored by both turbidity measurement (optical density determination) at 350 nm and size exclusion high performance liquid chromatography (SE-HPLC). In another experiment, the samples were incubated at 50 °C (up to 240 h) and analyzed by SE-HPLC, optical density determination, dynamic light scattering and electrophoresis techniques. The relationship between thermal stability of protein with pH of sodium phosphate buffer solution (5, 6, and 7) and buffer concentrations (10, 55, and 100 mM) were also studied through heating the protein samples (100 μg/mL) at 50 °C for 72 h by SE-HPLC.

*Turbidity measurements*

Optical density of the protein sample was determined by ScanDrop^®^ 250 (Analytik Jena AG, Jena, Germany) at 350 nm against sodium phosphate buffer as the blank solution to evaluate the aggregate production. 

*Size exclusion high performance liquid chromatography (SE-HPLC)*

Size exclusion separation was carried out on a TSKgel G3000 SWXL column (7.8 mm ID×300 mm, 5-μm particles, 250Å pore size, Tosoh Bioscience, Japan). Analysis was performed at 25 °C under isocratic elution at a rate of 0.5 mL/min with a mobile phase consisting of 100 mM sodium phosphate (pH 7.0), 150 mM NaCl and 0.05% (w/v) sodium dodecyl sulfate (SDS).The column was calibrated by protein standard markers.

*Sodium dodecyl sulfate polyacrylamide gel electrophoresis (SDS-PAGE)*

Gels were composed of a separating gel containing 12.5% (w/v) acrylamide and 0.1% (w/v) SDS, and a stacking gel containing 3% (w/v) acrylamide and 0.1% (w/v) SDS. Gels were run under reducing (sample buffer containing 5% v/v β-mercaptoethanol) and non-reducing (sample buffer without β-mercaptoethanol) conditions at 200 V at room temperature, using a MP300 electrophoresis instrument (Cleaver Scientific, UK). 

*Dynamic light scattering (DLS)*

A Malvern Zetasizer Nano ZS (Worcestershire, UK) laser light scattering system, equipped with a Nano ZS^®^ software for data acquisition and analysis, was used for particle size measurements. 

*Statistical analysis*

Results are reported as mean ± SD. Data obtained was compared using one-way ANOVA. Differences between the treatments were assumed to be significant at p < 0.05. Statistical analysis of all data was performed using IBM^®^ SPSS^®^ Statistics, v19, 2010.

## Results and Discussion

*Heating stress studies*

**Figure 1:**
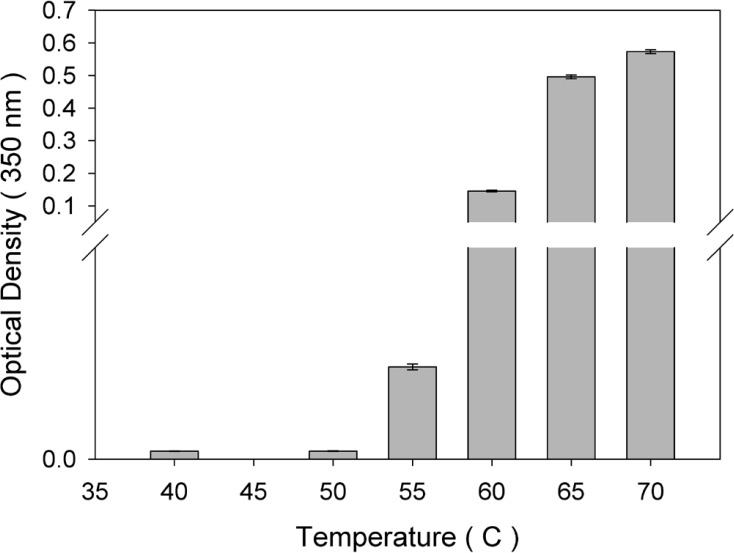
Optical density of rhIFNα2b solutions (100 μg/mL) following incubation at various temperatures for 1 h (mean ± SD; n = 3).

An increase in optical density signal in the non-absorbing region of a protein UV spectrum (320 nm) is typically considered as an indicator of larger species of aggregate forms (19, 20).In this study, the optical density of protein samples incubated at various temperatures for 1 h was monitored (Figure 1). As depicted, compared with non-incubated rhIFNα2b, no significant change in optical density was observed when incubation was performed at 40 and 50 °C, whereas at temperatures higher than 50 °C, optical density increased significantly.

**Figure 2:**
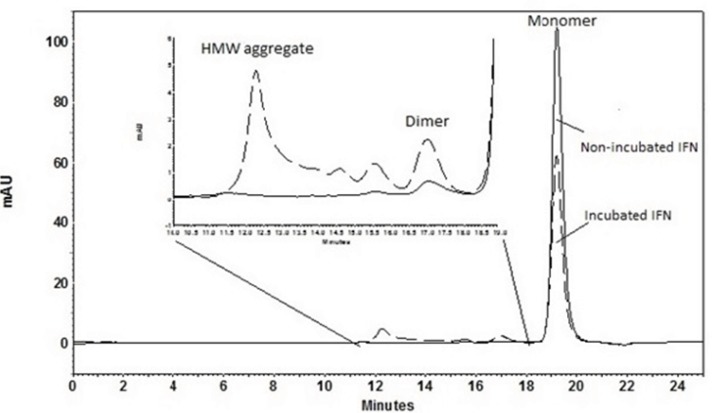
SE-HPLC chromatograms of non-incubated rhIFNα2b and a sample incubated at 50 °C for 240 h.

**Figure 3:**
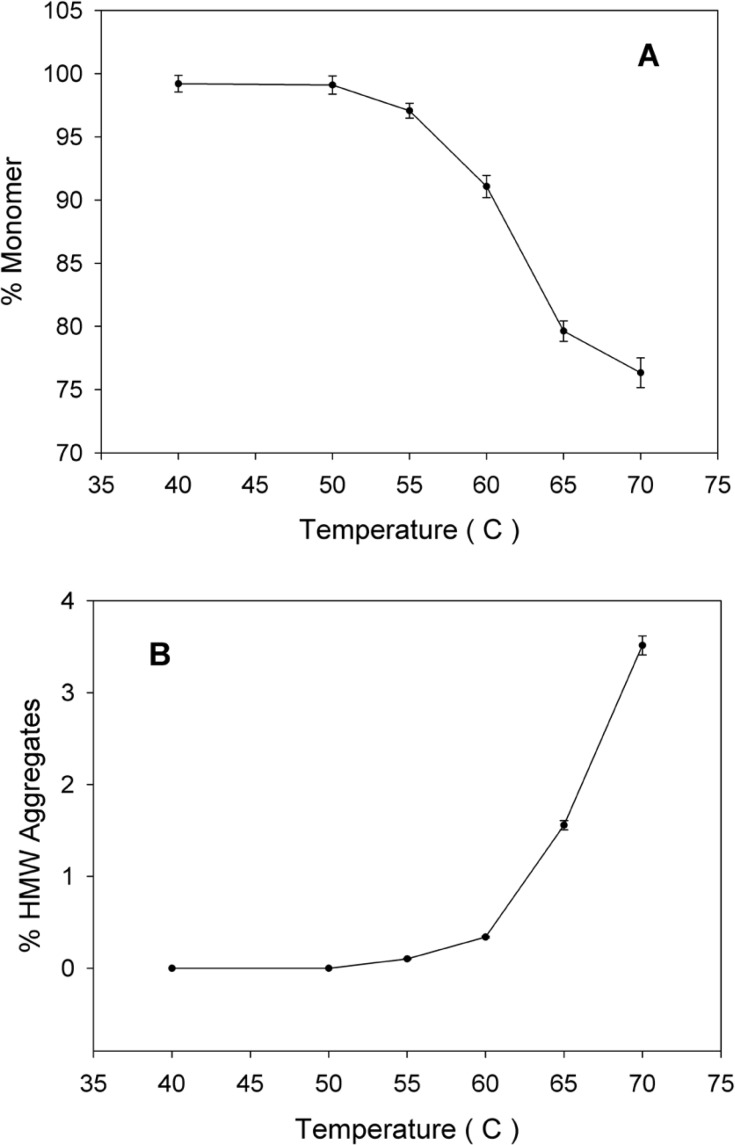
A) Plots of percent of monomer and B) percent of high molecular weight aggregates, following incubation of rhIFNα2b solutions (100 μg/mL) for 1 h at various temperatures (mean ± SD; n = 3).

**Figure 4:**
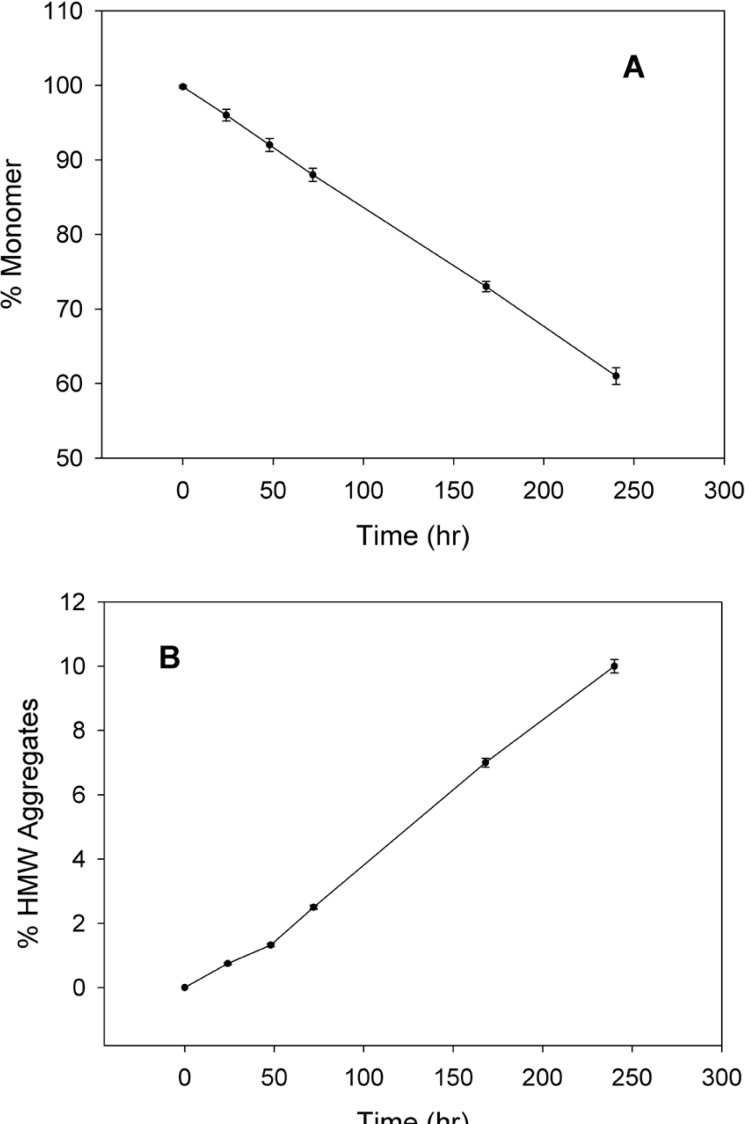
Plots of A) percent of monomer, B) percent of high molecular weight aggregates, and C) optical density as a function of time, following incubation of rhIFNα2b solutions (100 μg/mL) at 50 °C (mean ± SD; n = 3).

SE-HPLC is one of the few analytical techniques suitable for the quantitative assessment of proteins in their native state and for the determination of their oligomers and aggregates (21, 22). Non-incubated rhIFNα2b used in this investigation, contained 99.8% monomer and 0.2% dimer. However, incubation was shown to increase the content of high molecular weight (HMW) aggregates and dimers. Figure 2 indicates SE-HPLC chromatograms of non-incubated rhIFNα2b and a sample incubated at 50 °C for 240 h. Plots of the percent of remained rhIFNα2b monomer and HMW aggregates as a function of incubation temperature are shown in Figure 3A and Figure 3B, respectively. Compared with non-incubated rhIFNα2b, incubation at temperatures greater than 50 °C for 1 h resulted in significant changes in the contents of monomer and HMW aggregates. Results obtained from optical density measurements and SE-HPLC for incubated rhIFNα2b at 50 ⁰C for 24, 48, 72, 168 and 240 h, also revealed a significant drop in the monomer content and a significant rise in the amount of HMW aggregate and OD_350_, as the incubation time increased (Figure 4).

**Figure 5:**
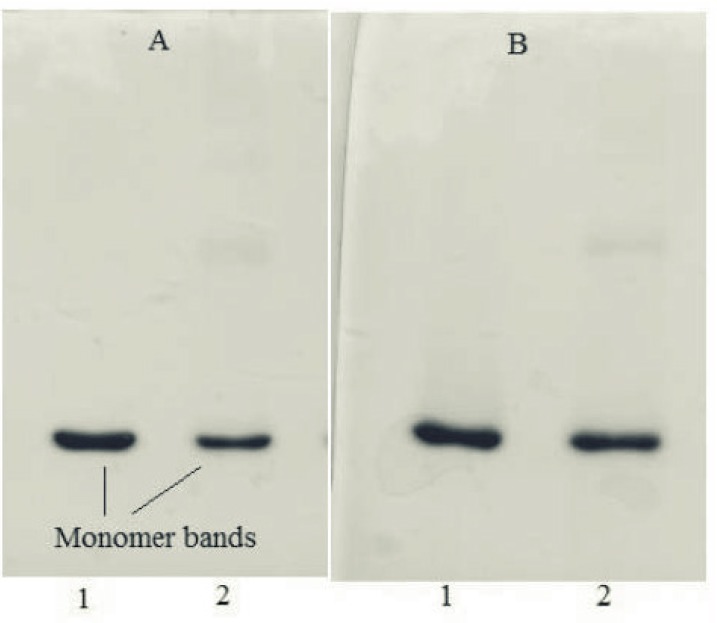
SDS–PAGE results of 1) non-incubated rhIFNα2b, and 2) incubated rhIFNα2b at 50 °C for 240 h, under non-reducing (A) and reducing (B) conditions

**Figure 6:**
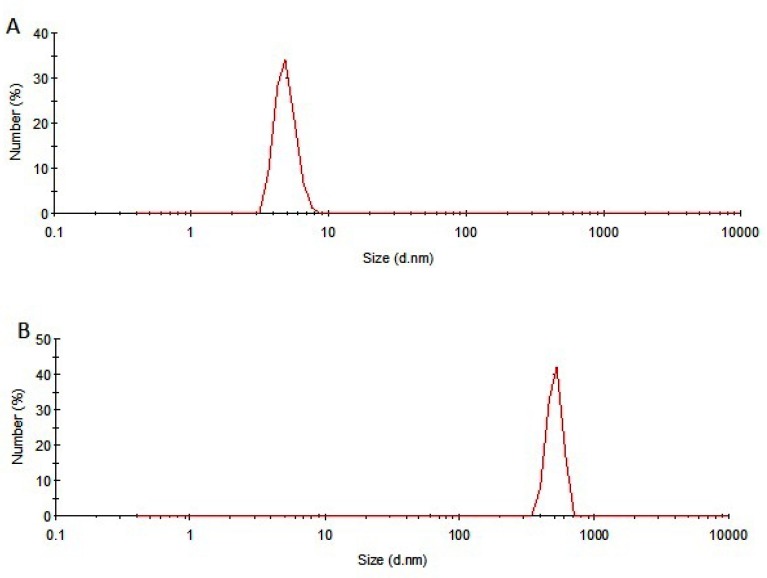
Size distribution of A) non-incubated and B) incubated rhIFNα2b at 50 °C for 240 h.

SDS-PAGE results of samples incubated at 50 °C for 240 h were in consistent with those obtained from other analyses. Under non-reducing condition, the monomer band of incubated rhIFNα2b sample was weaker than the non-incubated one (Figure 5A), whereas at reducing condition, this band showed higher intensity (Figure 5B) which may be possibly due to the formation of intermolecular disulfide bonds in thermally induced aggregates. DLS analysis also revealed the presence of aggregates (heterogeneous in size) in the sample incubated at 50 ⁰C for 240 h. Figure 6 depicts that the size distribution peak shifted to the large particles for incubated samples.

*Effect of pH and buffer concentration*

Theoretically, a protein has minimum solubility at its pI due to the minimum protein charge-charge repulsions. Nevertheless, the rate and extent of protein aggregation were expected to be maximal around the pI and negatively correlated with the absolute value of charge (23, 24). Militello *et al. *found that the aggregation of bovine serum albumin increased as the pH was adjusted closer to its pI (about 5) at 58 °C (25). Giger *et al. *studied the aggregation of insulin (with pI of 5.5) in 10 mM NaCl solution at room temperature in the pH range of 3-9 by turbidity measurement and found that this process was fastest at pH 5.6 (26). However, this is certainly not the case for all proteins. Some proteins actually aggregate at a lower rate around their pI values. Majhi *et al*. (27) found that the initial aggregation rate of β-lactoglobulin at the concentration of 1 mg/mL in 4.5 mM NaCl is bell shaped at ambient temperature with a maximum rate at pH 4.6-4.7. They also observed that the aggregation rate at the pI point (pH 5.2) was 30 times lower than that at pH 4.7. Similarly, non-native aggregation of recombinant human granulocyte colony stimulating factor in phosphate buffer solution upon incubation at 37 °C for 5 days was found to take place easily at pH 6.9 (with 96% loss of monomer), but only 30% is lost at pI (pH 6.1) (28). In this research, we studied the aggregate formation of rhIFNα2b at its pI (about 6) and evaluated the effect of pH 5 and 7 on the protein aggregation. The results confirmed that following 72 h incubation at 50 ⁰C, the percentage of monomer was significantly higher and the percentage of HMW aggregates was significantly lower, at pH 6 compared to pH 7 and pH 5 in all buffer concentrations (Table 1).

Buffer concentrations can also influence the aggregation behavior of proteins to a great extent (29-32). It has been reported in the literature that by increasing the concentrations of both phosphate and citrate buffers from 0 to 80 mM, the aggregation rate of recombinant human interleukin -1 receptor antagonist at pH 6.5 in the presence of 80 mM NaCl was reduced (33). In the present study, the effect of different concentrations of phosphate buffer on the aggregation behavior of rhIFNα2b incubated for 72 h at 50 °C was evaluated. Data obtained by SE-HPLC technique revealed that irrespective of the pH applied, the lowest percentage of dimer and HMW aggregates (and in turn the highest percentage of monomer) was produced at the buffer concentration of 100 mM (Table 1). Data presented in Table 1 indicates that approximately 8% of the protein was not detected by SE-HPLC method following incubation which may be due to the surface adsorption of protein and to a lesser extent, the production of non-detectable, very high molecular weight aggregates during incubation.

**Table 1:** Effects of pH and buffer concentration on heat-induced aggregation of rhIFNα2b after 72 h incubation at 50°C (Mean ± SD; n = 3).

**pH**	**5**			**6**			**7**			
**Buffer conc. (mM)**	**10**	**55**	**100**	**10**	**55**	**100**	**10**	**55**	**100**
% monomer	84.6 ± 0.65	85.2 ± 0.38	87.5 ± 0.50	88.1 ± 0.55	88.7 ± 0.85	91.1 ± 1.36	85.7 ± 0.87	86.2 ± 0.72	88.6 ± 0.56
% dimer	2.5 ± 0.10	2.3 ± 0.09	1.7 ± 0.06	1.44 ± 0.22	1.2 ± 0.06	0.76 ± 0.04	1.79 ± 0.08	1.59 ± 0.08	1.23 ± 0.06
% aggregates	4.1 ± 0.10	3.9 ± 0.10	2.7 ± 0.08	2.4 ± 0.05	2.3 ± 0.05	0.95 ± 0.05	3.64 ± 0.05	3.5 ± 0.1	2.4 ± 0.1

## Conclusions

Due to the poor stability, proteins are susceptible to physical degradation, specifically aggregation, affecting the efficacy and safety of the biomolecules. To stabilize the protein against aggregation, solution conditions should be carefully chosen and controlled during various processes. The results obtained in this study indicate that thermal stress mediates rhIFNα2b aggregation. Our observations supported a pH and buffer concentration-dependent model for heat-induced aggregation behavior of rhIFNα2b solutions and confirmed that both factors could diminish the aggregation of the protein during thermal stress. Understanding the effects of such factors would play a key role in the protein stabilization and development of therapeutic protein formulations.

## References

[1] Aggarwal S (2008). What’s fueling the biotech engine-2007. Nat. Biotechnol.

[2] Wang W, Singh S, Zeng DL, King K, Nema S (2007). Antibody structure, instability and formulation. J. Pharm. Sci.

[3] Gokarn YR, Kras E, Nodgaard C, Dharmavaram V, Fesinmeyer RM, Hultgen H, Brych S, Remmele RL, Jr Brems DN, Hershenson S (2008). Self-buffering antibody formulations. J. Pharm. Sci.

[4] Carpenter JF, Cherney B, Rosenberg AS, Mahler HC, Jiskoot W (2012). The critical need for robust assays for quantitation and characterization of aggregates of therapeutic proteins. Analysis of aggregates and particles in protein pharmaceuticals.

[5] Nafissi-Varcheh N, Aboofazeli R (2011). An approach to the design of a particulate system for oral protein delivery.II . Preparation and stability study of rhGH-loaded in simulated gastrointestinal fluids. Iran. J. Pharm. Res.

[6] Sharma VK, Kalonia DS (2003). Temperature- and pH-induced multiple partially unfolded states of recombinant human interferon-alpha2a: Possible implications in protein stability. Pharm. Res.

[7] Ablinger E, Hellweger M, Leitgeb S, Zimmer A (2012). Evaluating the effects of buffer conditions and extremolytes on thermostability of granulocyte colony-stimulating factor using high-throughput screening combined with design of experiments. Int. J. Pharm.

[8] Eppler A, Weigandt M, Hanefeld A, Bunjes H (2010). Relevant shaking stress conditions for antibody preformulation development. Eur. J. Pharm. Biopharm.

[9] Nafissi-Varcheh N, Luginbuehl V, Aboofazeli R, Merkle HP (2011). Preparing poly (lactide-co-glycolic acid) (PLGA) microspheres containing lysozyme-zinc precipitate using a modified double emulsion method. Iran. J. Pharm. Res.

[10] Beldarrain A, Cruz Y, Cruz O, Navarro M, Gil M (2001). Purification and conformational properties of a human interferon alpha2b produced in Escherichia coli. Biotechnol. Appl. Biochem.

[11] Gitlin G, Tsarbopoulos A, Patel ST, Sydor W, Pramanik BN, Jacobs S, Westreich L, Mittelman S, Bausch JN (1996). Isolation and characterization of a monomethioninesulfoxide variant of interferon alpha- 2b. Pharm. Res.

[12] Bordens R, Grossberg SE, Trotta PP, Nagabhushan TL (1997). Molecular and biologic characterization of recombinant interferon-alpha2b. Semin. Oncol.

[13] Yuen P, Kline D (1998). Stable aqueous alpha interferon solution formulations. US Patent.

[14] Isaacs A, Lindenmann J (1957). Virus interference. I. The interferon. Proc. R. Soc. Lond. B. Biol. Sci.

[15] Streuli M, Nagata S, Weissmann C (1980). At least three human type alpha interferons: Structure of alpha 2. Science.

[16] Ruiz L, Reyes N, Duany L, Franco A, Aroche K, Hardy Rando E (2003). Long-term stabilization of recombinant human interferon alpha 2b in aqueous solution without serum albumin. Int. J. Pharm.

[17] Braun A, Kwee L, Labow MA, Alsenz J (1997). Protein aggregates seem to play a key role among the parameters influencing the antigenicity of interferon alpha (IFN-alpha) in normal and transgenic mice. Pharm. Res.

[18] Hermeling S, Aranha L, Damen JM, Slijper M, Schellekens H, Crommelin DJ, Jiskoot W (2005). Structural characterization and immunogenicity in wild-type and immune tolerant mice of degraded recombinant human interferon alpha2b. Pharm. Res.

[19] Kueltzo LA, Midddaugh CR, Jiskoot W, Crommelin DJA (2005). Ultraviolet absorption spectroscopy. Methods for structural analysis of protein pharmaceuticals.Biotechnology: Pharmaceutical aspects.

[20] Esfandiary R, Middaugh CR, Mahler HC, jiskoot W (2012). ultraviolet absorption spectroscopy. Analysis of aggregation and particles in protein pharamaceuticals.

[21] Qian J, Tang Q, Cronin B, Markovich R, Rustum A (2008). Development of a high performance size exclusion chromatography method to determine the stability of human serum albumin in a lyophilized formulation of interferon alfa-2b. J. Chromatogr. A.

[22] Sluzky V, Shahrokh Z, Stratton P, Eberlein G, Wang YJ (1994). Chromatographic methods for quantitative analysis of native, denatured, and aggregated basic fibroblast growth factor in solution formulations. Pharm. Res.

[23] Chiti F, Stefani M, Taddei N, Ramponi G, Dobson CM (2003). Rationalization of the effects of mutations on peptide and protein aggregation rates. Nature.

[24] DuBay KF, Pawar AP, Chiti F, Zurdo J, Dobson CM, Vendruscolo M (2004). Prediction of the absolute aggregation rates of amyloidogenic polypeptide chains. J. Mol. Biol.

[25] Militello V, Casarino C, Emanuele A, Giostra A, Pullara F, Leone M (2004). Aggregation kinetics of bovine serum albumin studied by FTIR spectroscopy and light scattering. Biophys. Chem.

[26] Giger K, Vanam RP, Seyrek E, Dubin PL (2008). Suppression of insulin aggregation by heparin. Biomacromol.

[27] Majhi PR, Ganta RR, Vanam RP, Seyrek E, Giger K, Dubin PL (2006). Electrostatically driven protein aggregation: beta-lactoglobulin at low ionic strength. Langmuir.

[28] Chi EY, Krishnan S, Kendrick BS, Chang BS, Carpenter JF, Randolph TW (2003). Roles of conformational stability and colloidal stability in the aggregation of recombinant human granulocyte colony-stimulating factor. Protein. Sci.

[29] Eberlein GA, Stratton PR, Wang YJ (1994). Stability of rhbFGF as determined by UV spectroscopic measurements of turbidity. PDA. J. Pharm. Sci. Technol.

[30] Paborji M, Pochopin NL, Coppola WP, Bogardus JB (1994). Chemical and physical stability of chimeric L6, a mouse-human monoclonal antibody. Pharm. Res.

[31] Pikal MJ, Dellerman KM, Roy ML, Riggin RM (1991). The effects of formulation variables on the stability of freeze-dried human growth hormone. Pharm. Res.

[32] Wang YJ, Shahrokh Z, Vemuri S, Eberlein G, Beylin I, Busch M (1996). Characterization, stability, and formulations of basic fibroblast growth factor. Pharm. Biotechnol.

[33] Raibekas AA, Bures EJ, Siska CC, Kohno T, Latypov RF, Kerwin BA (2005). Anion binding and controlled aggregation of human interleukin-1 receptor antagonist. Biochem.

